# Brain correlates of perceived cognitive impairment after covid-19 infection: a multimodal MRI study.

**DOI:** 10.1192/j.eurpsy.2023.319

**Published:** 2023-07-19

**Authors:** V. Bettonagli, M. Paolini, M. Palladini, M. G. Mazza, P. Rovere Guerini, S. Poletti, F. Benedetti

**Affiliations:** 1 Division of Neuroscience, In vivo structural and molecular neuroimaging unit, Vita-Salute San Raffaele University Milan, Italy; 2 Division of Neuroscience, In vivo structural and molecular neuroimaging unit, IRCCS San Raffaele Scientific Institute, Milan, Italy

## Abstract

**Introduction:**

Many different long-term neuropsychiatric sequelae of the novel Coronavirus have been described after the pandemic outbreak. One of the most common symptoms in the months following infection is related to “brain fog”. This condition includes several signs of cognitive impairment like mental slowness, deficits in attention, executive functions, processing, memory, learning, and/or psychomotor coordination, which can be perceived on a subjective level and further confirmed by objective data. Since this kind of mental status has been documented in previous viral infections, and the SARS-COV-2 has been characterized by a worldwide diffusion, investigation into this condition in post-covid individuals is warranted. Currently, several hypotheses on its pathophysiology have been put forward, mostly hypothesizing a direct effect of the virus on the central nervous system or indirect consequences of the inflammatory response.

**Objectives:**

The aim of our research is to analyze brain correlates of subjective cognitive complaints in Covid-19 survivors using multimodal brain imaging.

**Methods:**

We performed a voxel-based morphometry (VBM) and a resting state functional connectivity analysis on 60 post-COVID-19 individuals recruited from the San Raffaele Hospital in Milan, that underwent a 3 tesla MRI scan. We assessed the perceived cognitive impairment both after the infection and at the time of the MRI scan through the PROMIS Cognitive Abilities scale. The difference of the two scores (delta PROMIS) was calculated as a measure of cognitive improvement over time.

**Results:**

We found the perceived amelioration of cognitive abilities (delta PROMIS) to be positively associated to grey matter volumes in the bilateral caudate, putamen and pallidum (pFWE: ˂0.001). Moreover, in the resting state fMRI analysis, subjective cognitive status at MRI was found to be associated with functional connectivity between the right putamen and pallidum, and two clusters belonging to the attentional (pFWE: ˂0.001) and salience (pFWE: 0.02) networks.

**Conclusions:**

This is one of the first studies investigating brain correlates of subjective cognitive impairment after COVID-19 infection; our main finding is the convergence of structural and functional results on brain areas located within the basal ganglia, implying their possible role in the pathophysiology of the condition. Moreover, this research could be interpreted as the first step toward understanding a very complex condition, with potential implications for the development of treatment and neurorehabilitative strategies.

**Disclosure of Interest:**

None Declared

